# Servant Leadership in Japan: A Validation Study of the Japanese Version of the Servant Leadership Survey (SLS-J)

**DOI:** 10.3389/fpsyg.2020.01711

**Published:** 2020-09-02

**Authors:** Yuka Kobayashi, Kazuhiro Watanabe, Yasumasa Otsuka, Hisashi Eguchi, Norito Kawakami, Kotaro Imamura, Dirk van Dierendonck

**Affiliations:** ^1^Department of Mental Health, Graduate School of Medicine, The University of Tokyo, Tokyo, Japan; ^2^Faculty of Human Sciences, University of Tsukuba, Tokyo, Japan; ^3^Department of Public Health, Kitasato University School of Medicine, Sagamihara, Japan; ^4^Rotterdam School of Management, Erasmus University Rotterdam, Rotterdam, Netherlands

**Keywords:** servant leadership, Japan, measurement, work engagement, affective commitment, well-being

## Abstract

**Purpose:**

The purpose of this study is to develop and validate a Japanese version of the Servant Leadership Scale and to clarify the relationship between servant leadership (SL) and well-being among Japanese workers.

**Methods:**

After the Japanese version of the SLS (SLS-J) and of its short form (SLS-J-short) were developed in conformity with the guidelines (Wild et al., 2005), a web-based survey was administered to 516 Japanese employees (20 or older and have a supervisor). Confirmatory factor analysis (CFA) was conducted to evaluate a construct validation of the SLS-J and the SLS-J-short. Convergent validity was estimated with theoretically related constructs (e.g., transformational leadership, supervisory support, and interpersonal justice) and potential consequences of SL (e.g., affective commitment, work engagement, job satisfaction, organizational citizenship behavior (OCB), psychological distress, and work performance). Intraclass correlation coefficient (ICC) using the test-retest method was conducted with 104 of the initial respondents to assess internal consistency reliability. Additionally, the effects of SL on employees’ work engagement and the mediating role of employees’ affective commitment were estimated.

**Results:**

CFA confirmed that an eight-factor model (SLS-J) and a five-factor model (SLS-J-short) had the most satisfactory fits for the two scales with Japanese workers. Tests of convergent validity and reliability showed sufficiency for each of the dimensions of SLS-J and SLS-J-short. Additionally, it was revealed that SL has an impact on employees’ work engagement through a mediation of affective commitment at a cross-sectional level, and the indirect association between SL and work engagement *via* affective commitment remained afterward.

**Conclusion:**

SLS-J and SLS-J-short were confirmed to have good reliability and validity for Japanese workers. Also, this study found that SL has an important role in enhancing the engagement of workers.

## Introduction

The mental health of workers, both in terms of distress and in terms of engagement at work, has been a major focus of the health field (Nieuwenhuijsen et al., 2010; Theorell et al., 2015), but it is only recently that the role of the direct supervisor has gained more attention. However, given the potential impact of superiors on the mental health and well-being of their subordinates (Artz et al., 2017; Kuroda and Yamamoto, 2018), it is important to better understand the nature of that influence. In the leadership literature, the quality of the relationship between leaders and followers has been a popular topic (Yammarino et al., 2005). Leadership based on positive psychology emphasizing ethical and moral leader behavior has drawn considerable attention in association with public corporate scandals (e.g., Enron, Fannie Mae, Lehmann Brothers, Tyco, and WorldCom) (Hoch et al., 2018).

Among ethical and moral value-based leadership forms, servant leadership (SL) is distinctive in that it places the most emphasis on the growth of followers. With the involvement of servant leaders, followers will be healthier, wiser, freer, and more autonomous, and the followers thus benefit (or at least they are not harmed) (Greenleaf, 1977). The core characteristic of servant leaders is that they go beyond their self-interest and focus on fulfilling the needs of their followers (Liden et al., 2015; van Dierendonck, 2011). Thereby, followers are enabled to realize a shared vision through enhancing their well-being and functioning (Stone et al., 2004). Transformational leadership (TL) theory, the dominant theory of positive leadership since the 1980s, resembles servant leadership theory in that it emphasizes the personal growth of followers (Avolio et al., 2004). However, the purpose and the way they encourage followers’ personal grow differs between the two theories (Liden et al., 2008; van Dierendonck, 2011). Transformational leaders inspire followers toward the organizational goals and enable them to perform better through “individualized consideration,” “intellectual stimulation,” and “supportive behavior.” Servant leaders promote the realization of shared vision by creating conditions that enhance followers’ well-being and functioning through “humility,” “authenticity,” and “interpersonal acceptance.” Servant leaders focus on the psychological needs of followers as a goal in itself and trust followers to do what is necessary for the organization (Stone et al., 2004).

Previous empirical research has shown that SL behavior is positively linked to subordinates’ attitudinal outcomes, such as work engagement (De Clercq et al., 2014; van Dierendonck et al., 2014), job satisfaction (Mayer et al., 2008), and organizational commitment (Walumbwa et al., 2018), and behavioral outcomes, such as organizational citizenship behavior (OCB; Walumbwa et al., 2010; Bavik et al., 2017), voice behavior (Chughtai, 2016), and helping behavior (Zou et al., 2015), as well as performance outcomes, such as job performance (Schwarz et al., 2016) and team performance (Hu and Liden, 2011). Furthermore, a recent meta-analysis based on 130 independent studies has shown that SL has incremental predictive validity over other representative leadership approaches (i.e., transformational, authentic, and ethical leadership) for performance outcomes such as individual-level task performance, team-level task performance, and OCB (Lee et al., 2019).

The mediating mechanism of the positive impact on well-being, which is a meaningful feature of SL, has been verified. The impact of SL on work engagement has been shown to be mediated by follower need satisfaction (van Dierendonck et al., 2014), organizational identification, and psychological empowerment (de Sousa and van Dierendonck, 2014). It has been also shown that organizational justice (Mayer et al., 2008) and leader-member exchanges (Amah, 2018) mediate the relationship between SL and job satisfaction. The evidence thus indicates that SL has an important impact on attitudes, performance, and other behaviors of followers, and it also might have a significant influence on the well-being of followers.

There are mainly three measures of SL behavior that have gone through a rigorous process of construction and validation (Eva et al., 2019). Included among them is the Servant Leadership Survey (SLS; van Dierendonck and Nuijten, 2011), a multi-dimensional measure covering the essential aspects of SL (e.g., listening, empathy, healing, awareness, persuasion, conceptualization, foresight, stewardship, commitment to people’s growth, and building community; Spears, 1995). It has been verified in cross-cultural studies (Bobbio et al., 2012; Rodríguez-Carvajal et al., 2014; van Dierendonck et al., 2017). The SLS consists of 30 items that represent eight dimensions (Empowerment, Humility, Standing back, Stewardship, Authenticity, Accountability, Courage, and Forgiveness). These dimensions were selected as indicators of SL through an analysis of the SL literature and interviews with servant leaders and were verified with empirical studies. The contents of the eight dimensions are shown in Table 1. Additionally, in the Dutch developmental sample, a second-order exploratory factor analysis revealed three factors (van Dierendonck and Nuijten, 2011). The first of these three factors is labeled “leader.” This factor includes characteristics such as enabling followers to express their talents by setting clear goals, providing a meaningful work environment, and providing challenges and the necessary tools and conditions (empowerment, accountability, courage, stewardship) for success. The second factor, “servant,” relates to the aspect of servant attitude of allowing the employees to flourish (humility, standing back, and authenticity). The third factor is “forgiveness,” which involves an accepting, not punishing, attitude. Forgiveness involves looking upon errors as part of the job, mistakes as enhancing learning, and resentment as an attitude that impairs functioning. However, it should be noted that this classification was not confirmed in the United Kingdom sample used in the same article. Furthermore, the long version of the SLS has been supplemented by a short version consisting of 18 items that has shown strong cross-cultural factorial stability (van Dierendonck et al., 2017).

**TABLE 1:** The contents of eight dimensions of SLS.

Dimension		Quality of each dimension
Empowerment	(leader side)	a motivational concept that aims at fostering a pro-active, self-con?dent attitude among followers and gives them a sense of personal power through encouraging self-directed decision making, information sharing, and coaching for innovative performance.
Stewardship	(leader side)	the element that stimulates others to act in the common interest by setting the right example, and acts as role model taking responsibility for the larger institution instead of self-interest
Accountability	(leader side)	the element that holds others accountable for performance they can control. It ensures that people know what is expected of them, which is bene?cial for both employees and the organization. It is emphasized to be relevant to SL.
Courage	(leader side)	the element that dares to take risks and trying out new approaches to old problems. Courage is related to pro-active behavior and implies creating new ways, while it is essential for innovation and creativity.
Humility	(servant side)	the element arises from a proper understanding of one’s strong and weak points. It focuses on an attitude to know their limitation and therefore actively seek the contributions of others in order to overcome those limitations.
Standing back	(servant side)	the element that gives priority to the interest of others first and gives them the necessary support and credits. Standing back also shows retreating into the background when a task has successfully been accomplished.
Authenticity	(servant side)	the element that express the “true self,” being true to oneself and representing inner thought and feelings consistently.
Forgiveness		the element that concerns for others even when confronted with offences, arguments, and mistakes. It is important that people feel accepted, are free to make mistakes, and know that they will not be rejected, thereby creating an atmosphere of confidence.

*This table is based on van Dierendonck and Nuijten (2011) and van Dierendonck et al. (2017).*

Regarding the relationship between SL and workers’ well-being, the process of enhancing work engagement has been explored. Work engagement is “a positive, fulfilling work-related state of mind that is characterized by vigor, dedication, and absorption” (Schaufeli et al., 2002) related to low levels of anxiety and depression (Hakanen and Schaufeli, 2012; Innstrand et al., 2012) and absence due to illness (Rongen et al., 2014). Servant leaders are assumed to promote work engagement among followers by satisfying their followers’ needs and empowering them (de Sousa and van Dierendonck, 2014; van Dierendonck et al., 2014; Lee et al., 2019). Also, empirical studies have shown that SL causes affective commitment (Lapointe and Vandenberghe, 2018; Lee et al., 2019). Affective commitment is defined as “the employee’s emotional attachment to, identification with, and involvement in the organization” and motivation to stay with the organization (Meyer and Allen, 1991), which is linked to high job performance, a low rate of turnover cognition, and low voluntary absenteeism (Luchak and Gellatly, 2007). According to social exchange theory, servant leaders are assumed to increase the level of employees’ attachment to the organization through motivation stemming from the norm of reciprocity motivations to return favors in social relationships with the leader (Zhang et al., 2019). When employees experience SL, they show affective commitment, which is mediated by followers’ need satisfaction, leadership effectiveness (van Dierendonck et al., 2014), or organizational support (Zhou and Miao, 2014). Moreover, employees with high affective commitment are more willing than others to invest time and energy in their work, dedicate themselves to their work, and concentrate fully on their work, showing high work engagement (Rhoades et al., 2001; Poon, 2013; Rivkin et al., 2018). So, it is assumed that SL not only enhances employees’ work engagement directly but also enhances it through the mediation of affective commitment.

The original language of the SLS is Dutch, and the English version has been translated into several other languages (i.e., Portuguese, Icelandic, Italian, Finnish, German, Turkish, and Spanish). The SLS validity is confirmed through confirmatory factor analysis. Furthermore, the configural equivalence of the instrument was confirmed in eight European countries (van Dierendonck et al., 2017). This is encouraging for the European context and culture; however, the Asian culture in general and the Japanese language in particular, brings its challenges. It makes it essential that a Japanese translation of the SLS is studied to confirm its reliability and validity among Japanese workers. As the impact of cultural factors on the relationship between SL and its consequences has been pointed out (Liden, 2012; Mittal and Dorfman, 2012; Lee et al., 2019; Zhang et al., 2019), it is essential to validate the SLS also in a different cultural context, outside of Europe. In terms of cultural context (Hofstede et al., 2010), the relationship between SL and outcomes has been more explicitly shown in low power distance (Mittal and Dorfman, 2012; Lee et al., 2019; Zhang et al., 2019), individualistic cultures (Lee et al., 2019), low masculinity (Zhang et al., 2019), and low uncertainty avoidance (Mittal and Dorfman, 2012). Little is known about the impact of SL on personal and organizational outcomes in Japanese society, with its high masculinity, high uncertainty avoidance, moderately high power-distance, and collectivistic cultural context. Validating this scale in Japan and considering the relationship between each dimension of SL and outcomes is thus assumed to be an important and meaningful contribution to the SL research field.

The first aim of this study was therefore to evaluate the internal consistency reliability, convergent validity, and structural validity of a developed Japanese version of the SLS (SLS-J) and a short version of the SLS (SLS-J-short). The second objective was to confirm the positive impact of SL on workers’ engagement and the mediating role of employees’ affective commitment in Japanese culture using two-wave data.

## Materials and Methods

### Study Design

We developed the Japanese version of the SLS (SLS-J) in accordance with the guidelines specified in the International Society of Pharmacoeconomics and Outcomes Research (ISPOR) task force (Wild et al., 2005). The cross-sectional validation study using a web-based survey was conducted in Japan (Eguchi et al., 2019; Momotani and Otsuka, 2019; Watanabe et al., 2020).

Our study was based on the consensus-based standards for the selection of health measurement instruments (COSMIN) checklist (Mokkink et al., 2010). We hypothesized that the dimensions of the SLS-J will show good structural validity and internal consistency. Based on the results of the original study (van Dierendonck and Nuijten, 2011) and previous empirical studies, we hypothesized that the dimensions of the SLS-J would be positively correlated with transformational leadership (rs ≧0.50) (van Dierendonck et al., 2014; Hoch et al., 2018), supervisory support (rs ≧0.45) (Yang et al., 2018), and supervisor interpersonal justice (rs ≧0.50) (Mayer et al., 2008). Also, we hypothesized that the dimensions of the SLS-J would show good convergent validity with work engagement (rs ≧0.50) (van Dierendonck et al., 2014; Hoch et al., 2018), job satisfaction (rs ≧0.40) (Mayer et al., 2008; van Dierendonck and Nuijten, 2011), affective commitment (rs ≧0.35) (Bobbio et al., 2012; Zhou and Miao, 2014; Hoch et al., 2018), OCB (rs ≧0.40) (Walumbwa et al., 2010; Hoch et al., 2018), and work performance (rs ≧0.10) (van Dierendonck and Nuijten, 2011; Schwarz et al., 2016; Hoch et al., 2018). Furthermore, based on previous research that SL will affect the mental health of followers (van Dierendonck, 2011), we predicted that SL would be negatively correlated with psychological distress. We conducted a follow-up survey (Time 2) 4 weeks after the first survey (Time 1) to assess the test-retest reliability of the SLS-J and SLS-J-short.

Next, we verified the effects of SL on employee work engagement, and the mediating role of the affective commitment of employees. In order to confirm the causal relationships between SL and worker engagement, a work engagement scale was also administered at Time 2. The affective commitment of employees was thus measured at Time 1, and the work engagement of employees was measured at both Time 1 and Time 2. Data from those who responded at both Time 1 and Time 2 were used.

### Participants

Data were collected in September and October 2017. Of all workers who were registered as respondents for an Internet survey company, 516 workers were selected and completed a web-based questionnaire in order of entry to the questionnaire website. The Internet survey company that conducted this survey had access to more than 10 million potential participants, and recruited participants based on their demographic attributes. To ensure that the panel quality is equivalent to paper-based survey (Chang and Vowles, 2013), registration information is checked, as is answering behavior, the content of answer, and a digital behavior data (Macromil, 2019). The recruitment of participants was balanced by gender (258 men and 258 women) to take into account the potential influence of gender (Hogue, 2016). The inclusion criteria were as follows: (1) aged 20 or older, (2) employed at private companies, non-profit organizations or government agencies/public organizations, and (3) had a supervisor. The exclusion criteria were as follows: (1) self-employed, freelance, or part-time employee and (2) executive officer. Because the Internet survey company ceased the survey when the number of participants reached 103% of the target number of respondents (*N* = 500 in this study), the response rate could not be calculated. The sample size was set based on a power analysis. The follow-up survey was terminated when the number of responding workers reached 104 (1:1 by gender).

### Measures

#### SLS-J and SLS-J-Short

The servant leadership survey (SLS) is an eight-dimensional, 30-item survey (van Dierendonck and Nuijten, 2011). Sample items for each dimension were as follows: “My manager gives me the information I need to do my work well” (Empowerment; seven items), “My manager learns from criticism” (Humility, five items), “My manager keeps himself/herself in the background and gives credit to others” (Standing Back, three items), “My manager emphasizes the importance of focusing on the good of the whole” (Stewardship, three items), “My manager is open about his/her limitations and weaknesses” (Authenticity, four items), “My manager holds me responsible for the work I carry out” (Accountability, three items), “My manager takes risks even when he/she is not certain of the support from his/her own manager” (Courage, two items), and “My manager keeps criticizing people for the mistakes they have made in their work” (reverse-scored item, Forgiveness, three items). In addition, the original short-version scale (SLS-short) consisted of five factors across 18 items (van Dierendonck et al., 2017). Dimensions of the SLS-short included Empowerment (six items), Humility (three items), Authenticity (three items), Standing Back (three items), and Stewardship (three items).

We translated and developed the original SLS according to ISPOR guidelines. First, we obtained permission from the developers of the original SLS to translate the scale into Japanese (preparation section in ISPOR). After author (HE) and an external collaborator conducted forward translation independently, reconciliation, back translation, back translation review, harmonization, and cognitive debriefing were conducted. Back translation was conducted by a fluent English speaker from Japan who did not know the purpose of the study. The author (DD), who developed the original survey (SLS), checked the back-translated scale and made revisions in the back-translation review section. The cognitive debriefing was conducted for five fluent Japanese speakers who were majoring in health science. They were asked to complete the harmonized scale and revise the wording of any items they had difficulty understanding. In addition, three health care experts who did not know the purpose of the study adjusted the wording after translation. The full list of SLS-J items is provided in the Appendix.

The SLS-J and SLS-J-short had the same dimensions and scaling as the original scale. All items were rated on a 6-point Likert scale (1 = “Fully disagree” to 6 = “Fully agree”). The score for each dimension of the SLS-J and SLS-J-short was calculated by averaging the item scores. This scale was measured at both Time 1 and Time 2. The internal consistencies (Cronbach’s alpha) for each factor of SLS-J at Time 1 and Time 2 were as follows: Empowerment, α = 0.94 and α = 0.93; Humility, α = 0.88 and α = 0.88; Standing Back, α = 0.77 and α = 0.78; Stewardship, α = 0.74 and α = 0.76; Authenticity, α = 0.79 and α = 0.73; Accountability, α = 0.61 and α = 0.55; Courage, α = 0.77 and α = 0.69; Forgiveness, α = 0.70 and α = 0.61. for SLS-J, and Empowerment (short), α = 0.93 and α = 0.92; Humility (short), α = 0.84 and α = 0.87; and Authenticity (short), α = 0.71 and α = 0.63. Standing Back and Stewardship consisted of the same items as SLS-J.

#### Transformational Leadership

Transformational leadership was measured with five factors from the transformational leadership scale taken from the Multifactor Leadership Questionnaire (Avolio et al., 1995). Royalties were paid to the developers. The internal consistencies (Cronbach’s alpha) for each factor were as follows: Idealized Influence (Attributed) (e.g., “Instills pride in me for being associated with him/her”), α = 0.77; Idealized Influence (Behavior) (e.g., “Talks about their most important values and beliefs”), α = 0.82; Inspirational Motivation (e.g., “Talks optimistically about the future”), α = 0.89; Intellectual Stimulation (e.g., “Re-examines critical assumptions to question whether they are appropriate”), α = 0.86; Individual Consideration (e.g., “Spends time teaching and coaching”), α = 0.83; there were four items for each factor. All items were responded to on a 5-point Likert scale (0 = “Not at all” to 4 = “Frequently, if not always”). Each factor score was calculated by averaging the items. This scale was measured at Time 1.

#### Supervisor Support and Supervisor Interactional Justice

A subscale of the Brief Job Stress Questionnaire (BJSQ) (Inoue et al., 2014) was used to measure supervisor support and supervisor interactional justice (three items each). Sample items for supervisor support were “My supervisor is able to avoid being self-righteous,” “My supervisor treats me with kindness and consideration,” and “My supervisor responds to me with a sincere attitude” (α = 0.81). All items were responded to on a 4-point Likert scale (1 = “Disagree” to 4 = “Agree”). Samples of supervisor interactional justice were “How freely can you talk with your supervisor?,” “How reliable is your supervisor when you are troubled?,” and “How well will your supervisor listen to you when you ask for advice on personal matters?”(α = 0.84). All items were responded to on a scale from 1 (“Not at all”) to 4 (“Extremely”). This scale was measured at Time 1.

#### Affective Commitment

A subscale of the Organizational Commitment Scale developed by The Japan Institute for Labour Policy and Training (2003) was used to measure affective commitment. A sample item for affective commitment was “I am proud to be a member of this company.” Internal consistency was α = 0.85. The three items were responded to on a scale from 1 (“Not at all applicable”) to 5 (“Very applicable”). This scale was measured only at Time 1.

#### Work Engagement

Work engagement was assessed using the Japanese version (Shimazu et al., 2008) of the short form of the Utrecht Work Engagement Scale (UWES) (Schaufeli et al., 2006). The UWES includes three subscales that reflect the underlying dimensions of engagement: vigor, dedication, and absorption (three items for each). All of nine items were responded to on a 7-point Likert scale (0 = “Never” to 6 = “Always”). Because the validation study recommends that work engagement should be treated as a unitary construct owing to high correlations among the three components, in this study, the score was calculated by summing up the scores for the items. This scale was measured at both Time 1 (α = 0.96) and Time 2 (α = 0.96).

#### Job Satisfaction

The Japanese version of the Job Satisfaction Scale (Tanaka, 1998) developed by McLean (1979) was used. A sample item is “I am satisfied because my coworkers are cooperative,” and internal consistency was α = 0.92. All of six items were responded to on a 4-point Likert scale (1 = “No” to 4 = “Yes”). This scale was measured at Time 1.

#### Organizational Citizenship Behavior

Organizational citizenship behavior (OCB) was measured using the Japanese OCB scale developed by Tanaka (2002). A sample item from the Japanese OCB scale was “I’m willing to help with my colleagues’ troubles at work”; internal consistency was α = 0.94. The 33 items were responded to on a scale ranging from 1 (“Not at all”) to 5 (“Any time”). This scale was measured at Time 1.

#### Psychological Distress

The Japanese version of the K6 scale (Furukawa et al., 2008) developed by Kessler and his colleagues (Kessler et al., 2002) was used to measure extent of psychological distress. A sample item was “During the last 30 days, about how often did you feel worthless?”; internal consistency was α = 0.92. The six items were responded to on a 5-point scale (0 = “None of the time” to 4 = “All of the time”) and summed to calculate a total score; higher scores indicate higher psychological distress. This scale was measured at Time 1.

#### Work Performance

Work performance was measured using one item from a validated scale, the Japanese short version of the WHO Health and Work Performance Questionnaire (WHO-HPQ; Suzuki et al., 2014). The item was “How would you rate your overall job performance on the days you worked during the past 4 weeks (28 days)?,” and participants responded on a scale ranging from 0 (Worst performance) to 10 (Top performance). This scale was measured at Time 1.

#### Demographic Variables

In the online surveys, we measured sex, age, job types, occupation, business content, and industry to describe the study population.

#### Statistical Analyses

We conducted confirmatory factor analysis (CFA) to assess for structural validity using Robust Maximum Likelihood, and calculated omega coefficients for each dimension of the SLS-J and SLS-J-short. Internal consistency reliability was assessed with Cronbach’s alphas and intraclass correlation coefficients (ICC) using the test-retest method. Also, we conducted correlational analyses for convergent validity testing. The relationship between SLS-J and work engagement and the mediating role of affective commitment were examined with Structural Equation Modeling (SEM) using Robust Maximum Likelihood method. Also, we populated work engagement at Time 1 on this model to control for the stability of work engagement. The significance of the indirect associations (i.e., mediating effects) was tested by estimating asymmetric 95% confidence intervals using the bootstrap method (a total of 1,000 samplings). These analyses used the data that from the completers both surveys Time 1 and 2 (*N* = 104), and all participants (*N* = 516) with and without missing values using FIML (Full Information Maximum Likelihood). We used SPSS version 22 for reliability testing and convergent validity testing, the R psycho package for omega coefficients, Mplus version 7.4 for CFA and SEM.

#### Ethics Review and Approval

The study protocol was approved by the ethics committee of the Department of Medicine, The University of Tokyo, Japan [No. 11242-(4)]. We obtained informed consent from all participants. The study aims and assurance of protection of personal information were presented *via* instructions on the website, and participants responded to the questionnaire after consenting to participate.

## Results

Demographic characteristics of 516 participants and their supervisors are shown in Table 2. A plurality of the participants were clerks (40.5%) as job type, and the majority of the workers worked for private companies (77.1%). With regard to supervisors’ demographics, a majority was male (74.8%), the largest share were 50–59 years old (36.6%), and 32.6% were regular employees. Because the study employed a web-based survey, there were no missing values on any variables or items. The total score for the SLS-J (*M* = 3.53, SD = 0.70) was lower than for previous research that used the SLS (*M* = 4.10, SD = 1.04 for the Netherlands; *M* = 3.73, SD = 1.04 for the United Kingdom (van Dierendonck and Nuijten, 2011); *M* = 3.68, SD = 1.25 for Italy (Bobbio et al., 2012); *M* = 3.73, SD = 0.97 for Spain; *M* = 3.85, SD = 1.01 for Mexico; and *M* = 4.46, SD = 0.74 for Argentina (Rodríguez-Carvajal et al., 2014). Characteristically, only the Forgiveness score was high in Japan (*M* = 4.17, SD = 1.17) compared to the Netherlands (*M* = 3.87, SD = 1.05), United Kingdom (*M* = 2.81, SD = 1.33) and Italy (*M* = 3.33, SD = 1.09) (Data for Spanish-speaking countries are not shown). Table 3 shows descriptions and intercorrelations of the SLS-J dimensions and SLS-J-short dimensions.

**TABLE 2:** Demographic characteristics of participants and their supervisors.

		Baseline Survey (*N* = 516)				Follow-up Survey (*N* = 104)			
		n (%)		Mean (SD)		n (%)		Mean (SD)	
Gender									
	Male	258	(50.0)			52	(50.0)		
	Female	258	(50.0)			52	(50.0)		
Age				40.6	(10.43)			40.0	(9.6)
Job types									
	Management (more than the section manager)	50	(9.7)			16	(15.4)		
	Profession	72	(14.0)			11	(10.6)		
	Technical	79	(15.3)			21	(20.2)		
	Clerks	209	(40.5)			31	(29.8)		
	Service	64	(12.4)			11	(10.6)		
	Production skill position	24	(4.7)			7	(6.7)		
	Others	18	(3.5)			7	(6.7)		
Occupation									
	Civil servant	42	(8.1)			9	(8.7)		
	Employee (clerical)	209	(40.5)			37	(35.6)		
	Employee (technical)	113	(21.9)			28	(26.9)		
	Employee (others)	152	(29.5)			30	(28.8)		
Business content									
	Sales	64	(12.4)			14	(13.4)		
	Service	73	(14.1)			15	(14.4)		
	Planning	21	(4.1)			3	(2.9)		
	Office work	186	(36.0)			34	(32.7)		
	IT engineer	21	(4.1)			6	(5.8)		
	Research and development	33	(6.4)			6	(5.8)		
	Manufacture	43	(8.3)			11	(10.6)		
	Others	75	(14.5)			15	(14.4)		
Industry									
	Private company	398	(77.1)			80	(76.9)		
	Non-profit organization	62	(12.0)			15	(14.4)		
	Government agency or Public organization	56	(10.9)			9	(8.7)		

**TABLE 3:** Descriptions and intercorrelations of SLS-J and SLS-J-short dimensions (*N* = 516).

SLS-J Dimension		Range	No. of items	Mean	SD	1	2	3	4	5	6	7	8	9	10	11
1	Empowerment, long	1–6	7	3.51	1.14	1										
2	Empowerment, short	1–6	6	3.51	1.15	0.99**	1									
3	Humility, long	1–6	5	3.37	1.03	0.82**	0.81**	1								
4	Humility, short	1–6	3	3.28	1.07	0.78**	0.78**	0.96**	1							
5	Standing back	1–6	3	3.35	1.09	0.78**	0.78**	0.79**	0.75**	1						
6	Stewardship	1–6	3	3.46	1.04	0.79**	0.78**	0.77**	0.77**	0.70**	1					
7	Authenticity, long	1–6	4	3.49	0.99	0.72**	0.72**	0.71**	0.67**	0.66**	0.68**	1				
8	Authenticity, short	1–6	3	3.49	1.02	0.66**	0.65**	0.64**	0.60**	0.60**	0.61**	0.97**	1			
9	Courage	1–6	2	3.27	1.13	0.70**	0.69**	0.69**	0.69**	0.66**	0.74**	0.65**	0.60**	1		
10	Accountability	1–6	3	3.78	0.94	0.55**	0.54**	0.50**	0.47**	0.47**	0.54**	0.56**	0.54**	0.46**	1	
11	Forgiveness	1–6	3	4.06	1.09	0.17**	0.18**	0.09*	0.06	0.13**	−0.01	−0.05	−0.08	−0.05	−0.13**	1

**p < 0.01, *p < 0.05.

### Structural Validity of SLS-J and SLS-J-Short

The relative cut-off standards of goodness of model fit for CFA was interpreted as follows, Chi-Square value (χ2) would provide an insignificant result at *p* > 0.05, Comparative Fit Index (CFI) was acceptable at ≧0.90 and well fit at ≧0.95 (Hu and Bentler, 1999). Tucker Lewis Index (TLI) was recommended at ≧0.90 but suggested at ≧0.95 as the stronger criteria (Hooper et al., 2008). Standardized Root Mean Square Residual (SRMR) was acceptable at ≦0.08, and good fit at ≦0.05. Root Mean Square Error of Approximation (RMSEA) was acceptable at ≦0.07, and good fit at ≦0.06. The criteria for the combination of fit indices were TLI ≧0.96 and SRMR ≦0.09, RMSEA ≦0.06 and SRMR ≦0.09, or CFI ≧0.96 and SRMR ≦0.09 (Hu and Bentler, 1999). Akaike Information Criterion (AIC) and Bayesian Information Criterion (BIC) would be chosen with smaller models.

The results of the CFA are shown in Table 4. For the SLS-J, fit indices of the eight-factor model showed well fit for SRMR, RMSEA, and the fit index combination of them, and showed acceptable fit for CFI and TLI (χ2 (377) = 762.54, *p* < 0.01, CFI = 0.941, TLI = 0.932, SRMR = 0.051, RMSEA = 0.045 AIC = 42605.95, BIC = 43106.99). Also, the eight-factor model fit better than the three-factor model (“leader,” “servant,” and “forgiveness”) or the one-factor model. While the eight-factor model also fit better than the eight-factor model underlying second-order factor, the second order model showed acceptable fit [χ2 (398) = 896.72, *p* < 0.01, CFI = 0.924, TLI = 0.917, SRMR = 0.091, RMSEA = 0.049 AIC = 42761.58, BIC = 43173.46]. For the SLS-J-short, goodness of fit of the five-factor model turned out to be quite good [χ2 (125) = 178.62, *p* < 0.01, CFI = 0.986, TLI = 0.983, SRMR = 0.026, RMSEA = 0.029, AIC = 25139.1, BIC = 25410.861], showing better fit than the one-factor model or the five-factor model underlying second-order factor. Although a direct comparison is not possible between the SLS-J and SLS-J-short, the five-factor model for the SLS-J-short was a strong fit.

**TABLE 4:** Model fit index from confirmatory factor analysis of SLS-J and SLS-J-short.

									Comparison of model fit		
Model tested	χ^2^	df	CFI	TLI	SRMR	RMSEA	AIC	BIC	Δχ^2^	Δdf	p
**SLS-J**											
Eight-factor model	762.54	377	0.941	0.932	0.051	0.045	42605.953	43106.994	–	–	–
Three-factor model	993.40	402	0.91	0.902	0.057	0.053	42900.848	43295.736	230.864	25	<0.001
One-factor model	1271.34	405	0.868	0.858	0.066	0.064	43300.839	43682.989	508.801	28	<0.001
Eight-factor model underlying second-order factor	896.72	398	0.924	0.917	0.091	0.049	42761.583	43173.456	134.182	21	<0.001
**SLS-J-short**											
Five-factor model	178.62	125	0.986	0.983	0.026	0.029	25139.11	25410.861	–	–	–
One-factor model	323.62	135	0.95	0.943	0.036	0.052	25350.03	25579.32	145.005	10	<0.001
Eight-factor model underlying second-order factor	185.00	130	0.985	0.983	0.027	0.029	25140.307	25390.827	6.381	5	<0.001

*All p < 0.001; CFI, Comparative Fit Index; TLI, Tucker-Lewis Index; SRMR, Standardized Root Mean-square Residual; RMSEA, Root Mean Square Error of Approximation; AIC, Akaike’s Information Criterion; BIC, Bayesian Information Criterion.*

### Reliability of SLS-J and SLS-J-Short

Omega coefficients in the first survey for the SLS-J dimensions are as follows: Empowerment (ω = 0.94), Humility (ω = 0.89), Standing back (ω = 0.78), Stewardship (ω = 0.75), Authenticity (ω = 0.79), Courage (ω = 0.77), Accountability (ω = 0.63), and Forgiveness (ω = 0.72), while for the SLS-J-short the dimensions are as follows: Empowerment (ω = 0.94), Humility (ω = 0.84), and Authenticity (ω = 0.72) (Standing back and Stewardship are the same variables as for the SLS-J). The ICCs for each dimension ranged from 0.65 to 0.84 for SLS-J and ranged from 0.74 to 0.83 for the SLS-J-short (Table 5).

**TABLE 5:** Omega coefficient and descriptive statistics and test-retest reliability of SLS-J and SLS-J-short dimensions.

	Omega	Test (*N* = 104)		Retest (*N* = 104)		Difference test-retest		Cronbach’s α (*N* = 104)		ICC	[95%Cl]
	coefficient										
Dimensions	(*N* = 516)	Mean	SD	Mean	SD	Mean	SD	Test	Retest		
Empowerment, long	0.94	3.48	1.11	3.66	1.06	0.18	0.79	0.92	0.93	0.84	[0.77–0.89]
Empowerment, short	0.94	3.45	1.13	3.64	1.08	0.18	0.84	0.91	0.92	0.83	[0.75–0.89]
Humility, long	0.89	3.39	0.97	3.48	1.02	0.09	0.82	0.83	0.88	0.80	[0.70–0.86]
Humility, short	0.84	3.27	1.05	3.36	1.12	0.10	0.91	0.83	0.87	0.79	[0.69–0.86]
Standing back	0.78	3.36	1.04	3.50	1.10	0.14	0.98	0.73	0.78	0.74	[0.61–0.82]
Stewardship	0.75	3.48	1.06	3.58	1.11	0.10	0.87	0.72	0.76	0.81	[0.71–0.87]
Authenticity, long	0.79	3.63	0.92	3.73	0.92	0.09	0.73	0.71	0.73	0.81	[0.73–0.87]
Authenticity, short	0.72	3.70	0.99	3.73	0.96	0.03	0.78	0.68	0.63	0.81	[0.72–0.87]
Courage	0.77	3.34	1.17	3.37	1.14	0.03	1.12	0.69	0.69	0.70	[0.55–0.79]
Accountability	0.63	3.90	0.88	3.96	0.84	0.05	0.88	0.56	0.55	0.65	[0.48–0.76]
Forgiveness	0.72	4.17	1.17	4.05	0.98	−0.12	0.96	0.75	0.61	0.75	[0.64–0.83]

*ICC, intraclass correlation coefficients; CI, confidence interval for ICC.*

### Convergent Validity of SLS-J and SLS-J-Short

As a result of the convergent validity testing (Table 6), dimensions for the SLS-J and SLS-J-short showed similar correlations with other variables. In relation to the dimensions of transformational leadership, they showed high correlations with Empowerment (rs = 0.64–0.80), and medium correlations were found with Humility (rs = 0.56–0.69), Standing back (rs = 0.53–0.64), Stewardship (rs = 0.60–0.68), Authenticity (rs = 0.53–0.58), Accountability (rs = 0.57–0.62), and Courage (rs = 0.36–0.40). Forgiveness was only weakly correlated (rs = 0.06–0.20) were found. Supervisory support and interpersonal justice were highly correlated with each dimension of the SLS-J. There was a moderate correlation between SLS-J dimensions and work engagement, job satisfaction, affective commitment, and OCB and a somewhat low correlation with psychological distress and work performance.

**TABLE 6:** Convergent validity (r) of SLS-J and SLS-J-short dimensions (*N* = 516).

			Empowerment, long	Empowerment, short	Humility, long	Humility, short	Standing back	Stewardship	Authenticity, long	Authenticity, short	Courage	Accountability	Forgiveness
	**Mean**	**SD**											
Transformational leadership													
Idealized Influence (Attributed)	1.78	0.89	0.74**	0.73**	0.67**	0.66**	0.62**	0.68**	0.57**	0.50**	0.38**	0.60**	0.15**
Inspirational Motivation	1.62	0.89	0.64**	0.62**	0.56**	0.55**	0.54**	0.60**	0.55**	0.50**	0.36**	0.58**	0.06
Intellectual Stimulation	1.77	0.94	0.78**	0.76**	0.69**	0.68**	0.64**	0.68**	0.56**	0.49**	0.38**	0.62**	0.15**
Individual Consideration	1.74	0.96	0.80**	0.79**	0.66**	0.63**	0.63**	0.65**	0.58**	0.53**	0.40**	0.57**	0.20**
Idealized Influence (Behavior)	1.77	0.92	0.69**	0.68**	0.60**	0.59**	0.53**	0.65**	0.53**	0.46**	0.37**	0.57**	0.06
Supervisory support	2.44	0.72	0.69**	0.68**	0.59**	0.56**	0.59**	0.56**	0.56**	0.52**	0.39**	0.52**	0.22**
Interpersonal justice	2.68	0.73	0.75**	0.74**	0.70**	0.66**	0.66**	0.62**	0.59**	0.52**	0.40**	0.53**	0.28**
Work engagement	2.78	1.51	0.42**	0.42**	0.32**	0.32**	0.34**	0.37**	0.35**	0.32**	0.31**	0.35**	0.01
Job satisfaction	2.95	0.86	0.59**	0.60**	0.53**	0.49**	0.48**	0.46**	0.43**	0.39**	0.40**	0.36**	0.15**
Affective commitment	2.93	1.02	0.51**	0.51**	0.45**	0.43**	0.44**	0.47**	0.42**	0.38**	0.40**	0.35**	0.10*
OCB	3.38	0.62	0.38**	0.38**	0.35**	0.31**	0.30**	0.37**	0.40**	0.39**	0.30**	0.42**	−0.07
Psychological distress	7.73	6.12	−0.23**	−0.24**	−0.16**	−0.13**	−0.18**	−0.15**	−0.15**	−0.12**	−0.10*	−0.21**	−0.18**
Work performance	5.83	1.89	0.20**	0.20**	0.14**	0.13**	0.17**	0.18**	0.17**	0.16**	0.18**	0.28**	−0.02

**p < 0.01, *p < 0.05. OCB, organizational citizenship behavior.

### The Relationship Between SLS-J, Affective Commitment, and Work Engagement

**FIGURE 1:**
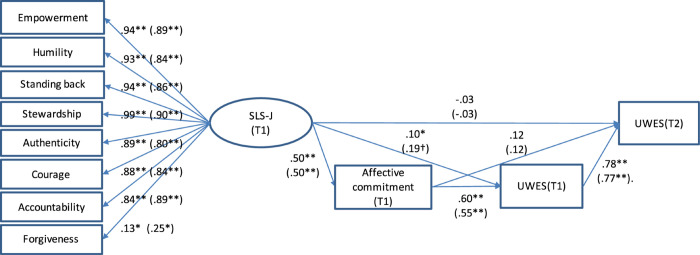
Structural equation modeling (SEM) results for the hypothesized model. ^∗∗^*p* < 0.01, ^∗^*p* < 0.05., ^†^*p* < 0.10. The hypothesized model using FIML (*N* = 516) is shown. Original data (*N* = 104) is shown in parentheses. T1 = Time 1, T2 = Time 2. UWES = Utrecht Work Engagement Scale. Hypothesized model using FIML (*N* = 516): (χ^2^ (485) = 1031.921, *p* < 0.01, CFI = 0.925, TLI = 0.919, RMSEA = 0.047, AIC = 45943.903, BIC = 46406.728. the standardized indirect effect from SLS-J to UWES(T2) = 0.30 (*p* < 0.01). Hypothesized model with original data (*N* = 104): (χ^2^ (485) = 814.314, *p* < 0.01, CFI = 0.796, TLI = 0.778, RMSEA = 0.081, AIC = 10130.969, BIC = 10419.208. the standardized indirect effect from SLS-J to UWES(T2) = 0.36 (*p* < 0.01).

Consistent with the hypotheses in this study, we confirmed the effect of SL on well-being and the mediating effect of affective commitment. Among the subjects (*N* = 104) who responded to both the survey at Time 1 and that at Time 2, work engagement at Time 2 showed *M* = 2.54, SD = 1.49. The correlations between work engagement and related variables were as follows: Empowerment (*r* = 0.38, *p* < 0.01), Humility (*r* = 0.22, *p* < 0.05), Standing back (*r* = 0.32, *p* < 0.01), Stewardship (*r* = 0.31, *p* < 0.01), Authenticity (*r* = 0.21, *p* < 0.05), Courage (*r* = 0.33, *p* < 0.01), Accountability (*r* = 0.28, *p* < 0.01), Forgiveness (*r* = −0.01, n.s.), and affective commitment (*r* = 0.62, *p* < 0.01). In the validation of the hypothesized model, the eight factors of the validated model were highly correlated with each other, and the estimation of path coefficients to the other variables (i.e., affective commitment and work engagement) was unstable due to multicollinearity. We therefore used the eight-factor model underlying the second-order factor as an alternative measure. In the initial model validation of dimensions of the SLS-J (Time 1) and work engagement (Time 2) using FIML (*N* = 516), SL had positive and significant association with work engagement (β = 0.43, *p* < 0.01). The model showed suboptimal fit [χ2 (426) = 880.669, *p* < 0.01, CFI = 0.932, TLI = 0.926, RMSEA = 0.045, AIC = 43045.013, BIC = 43473.870]. After adopting affective commitment and work engagement (Time 1) in the model as a mediator and a controller (Figure 1), the direct association between SL and work engagement (Time 2) became insignificant (β = −0.03, *p* = 0.68) but the indirect associations via affective commitment and work engagement (Time 1) was positive and significant (using FIML [*N* = 516], unstandardized coefficient: β = 0.45, 95%CI, 0.32 to 0.57, standardized coefficient: β = 0.30, 95%CI, 0.22 to 0.38). Factor loadings for SLS-J dimensions were generally adequate, except for Forgiveness. SL was positively associated with employees’ affective commitment (β = 0.49, *p* < 0.01). Also, employees’ affective commitment was associated with work engagement at Time 1 (β = 0.59, *p* < 0.01), and work engagement at Time 1 affected that of Time 2 (β = 0.78, *p* < 0.01).

## Discussion

In this study, we developed a Japanese version of the SLS (van Dierendonck and Nuijten, 2011) and of a short form of the SLS (van Dierendonck et al., 2017) and examined their reliability and construct validity. Our analyses showed that the SLS-J and SLS-J-short had good structural validity, reliability, and convergent validity.

Internal consistency and ICC were high and similar to the results for the original survey (SLS). For the dimension of Accountability, our results show relatively low reliability compared to the results of the original study. Accountability, giving followers responsibility, is an essential element of effective and positive leadership, with benefits including providing meaning and promoting self-determination (van Dierendonck and Nuijten, 2011). However, there may be a range in interpretation of the concept of accountability in Japan, where individuals tend to accept the ambiguity regarding where responsibility lies. This point should be clarified in further research.

The results of structural validity analyses supported the study hypotheses for both the SLS-J and SLS-J-short. The model fit index from confirmatory factor analysis indicated reasonable fit with the eight-factor model for the SLS-J and good fit with the five-factor model for the SLS-J-short, respectively. We believe that the eight-factor model (SLS-J), which is based on the theoretical background of servant leadership, and the five-factor model (SLS-J-short), which has been developed in consideration of cultural differences, are both important. In Japan, the results with respect to the SLS-J-short showed more suitability for practical use.

Convergent validity analyses supported our hypotheses, indicating that most of the SLS-J and SLS-J-short dimensions correlated significantly with relevant factors. Only Forgiveness was shown to be unrelated to Inspirational Motivation and Idealized Influence of Transformational leadership, Engagement, and OCB. Looking at the correlation trend, the high correlation with Empowerment on the one hand and the low correlation with Forgiveness, on the other hand, were similar to results reported in previous studies (van Dierendonck and Nuijten, 2011; Bobbio et al., 2012; van Dierendonck et al., 2017). The results that each dimension expect for Forgiveness showed medium correlation with work engagement and job satisfaction were also similar as in previous studies.

Apart from Forgiveness, the factor loadings of the SLS-J dimensions on the overall second-order SL dimension were mostly good. This is in line with previous international studies. Actually, Forgiveness was also the lowest factor loading in studies in Spain (0.18), Mexico (0.32) (Rodríguez-Carvajal et al., 2014), Italy (0.39) (Bobbio et al., 2012), and the Netherlands (0.19) (van Dierendonck and Nuijten, 2011). Nevertheless, since the eight-factor model has also been confirmed in each country, this may be due to negative formulation of the forgiveness items, whereas the other items are all formulated positively.

We also verified the relationship between SL and employees’ well-being in the Japanese cultural setting. This study revealed that employees’ work engagement was associated with SL with a mediation of affective commitment at a cross-sectional level, and the indirect association between SL and work engagement via affective commitment remained afterward. This is the first study to examine the effects of SL on workers’ well-being and the mediating role of workers’ affective commitment in the association between SL and work engagement in Japan, confirming its importance for Japanese culture.

Cultural context is assumed to be associated with SL effects (van Dierendonck, 2011; Eva et al., 2019). According to thorough analyses, SL has shown a stronger impact when the context involves low power distance, high individualism (Lee et al., 2019; Zhang et al., 2019), and low masculinity (Zhang et al., 2019) in terms of Hofstede’s cultural dimensions model (Hofstede et al., 2010). In this study, the SLS-J showed the lowest total score within The Netherlands, United Kingdom, Italy, and Spanish speaking countries, and the highest score of Forgiveness in The Netherlands, United Kingdom, and Italy in previous studies. In terms of Hofstede’s (2010) cultural dimensions, there are higher levels of masculinity, uncertainty avoidance, and collectivism in Japan than in Western countries (Hofstede et al., 2010). In Japan, one of the most masculine societies in the world, value has been placed on competition rather than feminine nurturance. This culture has led to high quality in manufacturing and services, but on the other hand, problems such as a long workday and obstacles to women’s social advancement exist. In this cultural context, the direction of the organization is emphasized, and the servant leadership style, which emphasizes employee need satisfaction, might be perceived as hard to evaluate. The higher Forgiveness score among our Japanese participants than those found in other countries may be explained by other features of Japanese culture: medium collectivism and high uncertainty avoidance. More collectivistic cultures are more forgiving than comparable individualistic cultures (Kadiangandu et al., 2001). It has been found that Japan has a collective corporate culture (Hofstede et al., 2010), and although competition among companies is fierce, human relationships within companies tend to be tolerant. In addition, Japan is often exposed to the threat of natural disasters such as earthquakes, tsunamis, and typhoons, and people have no choice but to accept an uncertain environment. Although it is pointed out that people in cultures with a high level of uncertainty avoidance generally have more difficulty in being forgiving (Neto and Pinto, 2010), people in Japan might value forgiveness in order to maintain social harmony as a way to deal with uncertainties such as natural disasters. Perhaps in Japan, regardless of who leaders are, a high level of forgiveness is thus required as a virtue.

Recently, it has been suggested that Japanese culture has become more individualistic and the coexistence of individualism and traditional collectivism would be related to undesirable interpersonal relationships (Ogihara, 2017; Takano and Osaka, 2018). The leadership style of listening humbly to each person’s perspectives, empowering them, and trying to empathize with other people would therefore become more important in this culture.

### Implications

This study has some useful implications for practitioners. First, this study confirmed that SL plays an important role in enhancing the work engagement of employees. Furthermore, measuring aspects of SL with the SLS-J made it possible to clarify the characteristics of leaders in Japan and understand the aspects that need to be strengthened to enhance their leadership abilities. Lastly, this study provides useful suggestions regarding the creation of a healthy workplace. As pointed out in previous studies (Lee et al., 2019; Zhang et al., 2019), organizations should implement, deploy, and support appropriate training development programs for leaders in order to establish reciprocal relationships with their followers and increase their commitment to the organization. The SLS-J and SLS-J-short will provide useful insight for that.

### Limitations

One of the limitations of this study is that we could not calculate the response rate because it was a web-based survey. This might cause a selection bias, so there is a possibility that it does not accurately reflect the population. According to a previous study in Japan, web-based survey participants were more highly educated after adjusting for age and gender (Tsuboi et al., 2012), and participants in this study were younger than the average age of Japanese citizens according to government statistics (Ministry of Health, Labour and Welfare, 2017). Also, the model fit with complete respondents to both surveys (*N* = 104) was not good, though the path coefficient from SLS-J to work engagement (Time 1) was larger than the analysis of using FIML. In the follow-up survey (Time 2), data was collected in order of entry until reaching 104 people, so there could be a selection bias and random missing values did not occur. Therefore, the analysis performed using FIML prevents the related overestimate and is a reasonable result.

Next, the main object of this survey was to validate the SLS-J, so many of the measurements were collected cross-sectional. Although the relationship between SLS-J and work engagement mediated by affective commitment was confirmed, there were limitations to establishing a temporal relationship mechanism due to few available variables.

The third limitation comes from all data being collected with subjective response forms. It might be necessary to pay attention to the possibility that questionnaire responses differ from workers’ actual attitudes or the supervisors’ behavior.

Finally, since our investigation was focused on the individual level, there is a possibility that the results may be different from those for the organization as a whole. Nevertheless, a previous meta-analysis study confirmed that SL is relevant to both team-level and individual-level performance and OCB (Lee et al., 2019). Moving forward, it will, of course, be important to take into consideration the multiple processes that affect these outcomes, such as procedural climate in relation to OCB (Walumbwa et al., 2010), and affective commitment and collective organizational commitment in relation to team-performance (Walumbwa et al., 2018) need to be considered. Therefore, further studies are required to address these limitations.

## Conclusion

In conclusion, the SLS-J and SLS-J-short were confirmed to have good reliability and validity for Japanese workers. Also, the SLS-J results showed that SL has a strong impact on employees’ work engagement which was mediated by affective commitment. The SLS-J and SLS-J-short could be useful for clarifying the characteristics of Japanese leaders as well as assessing the relationships between SL and relative outcomes in Japan.

## Data Availability Statement

The datasets generated for this study are available on request to the corresponding author.

## Ethics Statement

The studies involving human participants were reviewed and approved by the Ethics Committee of the Department of Medicine, The University of Tokyo, Japan [No. 11242-(4)]. The patients/participants provided their written informed consent to participate in this study.

## Author Contributions

YK, KW, YO, and HE contributed to the conception and design of the study. YK conducted the survey and wrote the first draft of the manuscript. The statistical analysis was performed by KW (CFA, reliability testing and SEM) and YK (reliability testing, convergent validity testing and SEM). YK, HE, YO, NK, and KI contributed to translation into Japanese. DD reviewed back translated items and counseled the study design. All authors read the manuscript critically and approved the submitted version of the manuscript.

## Conflict of Interest

The authors declare that the research was conducted in the absence of any commercial or financial relationships that could be construed as a potential conflict of interest.
